# Machine learning approach for automated localization of ventricular tachycardia ablation targets from substrate maps: development and validation in a porcine model

**DOI:** 10.1093/ehjdh/ztaf064

**Published:** 2025-06-10

**Authors:** Xuezhe Wang, Adam Dennis, Eva Melis Hesselkilde, Arnela Saljic, Benedikt M Linz, Stefan M Sattler, James Williams, Jacob Tfelt-Hansen, Thomas Jespersen, Anthony W C Chow, Tarvinder Dhanjal, Pier D Lambiase, Michele Orini

**Affiliations:** Institute of Cardiovascular Science, University College London, 1-19 Torrington Pl, London WC1E 7HB, UK; Institute of Cardiovascular Science, University College London, 1-19 Torrington Pl, London WC1E 7HB, UK; Department of Biomedical Sciences, Faculty of Health and Medical Sciences, University of Copenhagen, Copenhagen, Denmark; Department of Biomedical Sciences, Faculty of Health and Medical Sciences, University of Copenhagen, Copenhagen, Denmark; Department of Biomedical Sciences, Faculty of Health and Medical Sciences, University of Copenhagen, Copenhagen, Denmark; Department of Biomedical Sciences, Faculty of Health and Medical Sciences, University of Copenhagen, Copenhagen, Denmark; Department of Cardiology, Heart Centre Copenhagen University Hospital, Copenhagen, Denmark; Abbott Medical United Kingdom, Blythe Valley Park, Solihull, UK; Department of Cardiology, Heart Centre Copenhagen University Hospital, Copenhagen, Denmark; Department of Forensic Medicine, Faculty of Health and Medical Sciences, University of Copenhagen, Copenhagen, Denmark; Department of Biomedical Sciences, Faculty of Health and Medical Sciences, University of Copenhagen, Copenhagen, Denmark; Barts Heart Centre, Barts Health NHS Trust, London, UK; University of Warwick, University Hospital Coventry & Warwickshire, UK; Institute of Cardiovascular Science, University College London, 1-19 Torrington Pl, London WC1E 7HB, UK; Barts Heart Centre, Barts Health NHS Trust, London, UK; Institute of Cardiovascular Science, University College London, 1-19 Torrington Pl, London WC1E 7HB, UK; School of Biomedical Engineering & Imaging Sciences, King’s College London, UK

**Keywords:** Ventricular tachycardia, Catheter ablation, Machine learning, Signal features, Substrate mapping, Intracardiac electrogram

## Abstract

**Aims:**

The recurrence rate of ventricular tachycardia (VT) after ablation remains high due to the difficulty in locating VT critical sites. This study proposes a machine learning approach for improved identification of ablation targets based on intracardiac electrograms (EGMs) features derived from standard substrate mapping in a chronic myocardial infarction (MI) porcine model.

**Methods and results:**

Thirteen pigs with chronic MI underwent invasive electrophysiological studies using multipolar catheters (Advisor™ HD grid, EnSite Precision™). Fifty-six substrate maps and 35 068 EGMs were collected during sinus rhythm and pacing from multiple sites, including left, right, and biventricular pacing. Ventricular tachycardia was induced in all pigs, and a total of 36 VTs were localized and mapped with early, mid-, and late diastolic components of the circuit. Mapping sites within 6 mm from these critical sites were considered as potential ablation targets. Forty-six signal features representing functional, spatial, spectral, and time-frequency properties were computed from each bipolar and unipolar EGM. Several machine learning models were developed to automatically localize ablation targets, and logistic regressions were used to investigate the association between signal features and VT critical sites. Random forest provided the best accuracy based on unipolar signals from sinus rhythm map, provided an area under the curve of 0.821 with sensitivity and specificity of 81.4% and 71.4%, respectively.

**Conclusion:**

This study demonstrates for the first time that machine learning approaches based on EGM features may support clinicians in localizing targets for VT ablation using substrate mapping. This could lead to the development of similar approaches in VT patients.

## Graphical Abstract

**Graphical Abstract:**
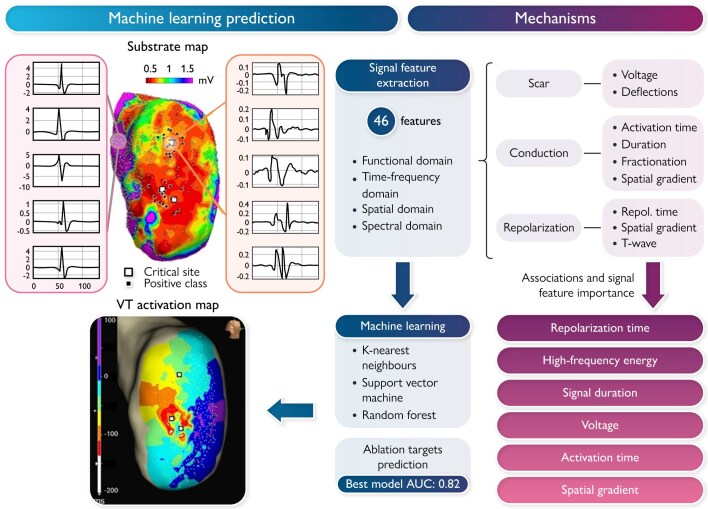

## Introduction

Ventricular tachycardia (VT) is a potentially life-threatening cardiac condition. Most of re-entrant VTs are caused by the tissue fibrosis which can slow or block the propagation of electrical signals, forming an arrhythmogenic substrate for a re-entry circuit(s).^1^ Catheter ablation is increasingly recommended as a strategy for treating VT,^2^ which has been proven with superior efficacy in maintaining sinus rhythm (SR) to anti-arrhythmic drug therapy alone.^3^

Although catheter ablation in VT has shown effective outcomes, its success rate remains unsatisfactory with more than 50% of patients experiencing recurrences within 1 year after ablation.^3^ This is mainly caused by the difficulty in accurately identifying the circuits during mapping and limitations in lesion delivery. Up to 90% of VTs may be haemodynamically unstable and require cardioversion or extracorporeal haemodynamic support, which limits the use of VT mapping for the delineation of the circuits.^4^

Substrate mapping has been proposed as an alternative method to predict VT-related tissue by analysing intracardiac electrograms (EGMs) without inducing VT.^5,6^ Numerous potential ablation targets were introduced including low voltage sites, late potentials, and functional parameters such as activation and repolarization time gradients (isochronal late activation mapping, decrement evoked potential, isochronal repolarization mapping, and re-entry vulnerability index), but it is challenging to combine different targets clinically.^7–10^

Artificial intelligence and machine learning could have value in this context through their ability to accurately solve complex classification problems. To date, it has shown significant potential in many aspects of electrocardiography,^11^ but its role in identification of VT ablation targets has been extremely limited. Only one study using multi-domain features with ensemble tree methods examined VT cases.^12^ However, that study focused on the identification of abnormal signal as opposed to critical sites of VT and potential ablation targets.

This study aims to develop and test machine learning models to localize critical components of VT circuits from data collected during substrate mapping and to determine the relationship between signal features and the VT critical circuit components. We established a porcine model of chronic myocardial infarction^13^ to enable accurate identification of VT circuits and to investigate multiple substrate mapping strategies.

## Methods

### Experimental study

The experimental protocol has been previously described.^13^ In brief, bipolar and unipolar endocardial EGMs were collected from 13 female Danish Landrace pigs (weight 42 ± 2 kg), with an infarct created by percutaneous coronary intervention.^14^ The EnSite Precision™ 3D cardiac mapping system (Abbott Medical, IL, USA) and the 16-electrode Advisor™ HD Grid Sensor Enabled Mapping Catheter (Abbott Medical, IL, USA) were used to create 3D electro-anatomical maps.

Up to six substrate maps were created by pacing from the left ventricle (LV), right ventricle (RV), and both (biventricular pacing, BIV). Sinus rhythm maps were created retrospectively from the intrinsic sinus template, from all recorded clinical segments. Two pacing protocols were performed, the sensed extra (SE) and programmed electrical stimulation (S1S2). The SE protocol refers to delivering an extra stimulation after a sinus beat, at least 20 ms after the ventricular effective refractory period (VERP). S1S2 represents a series of eight steady-state stimuli at a fixed rate (S1S1), followed by a stimulus delivered at a shorter coupling interval (S1S2).

Ventricular tachycardia was induced in all pigs and mapped to localize the diastolic pathway. Three VT critical sites were defined based on the occurrence of diastolic activity: early, mid-, and late diastolic points, indicating entry, isthmus, and exit of the VT circuit. Advanced signal filter in the mapping system was implemented to capture near-field EGMs. Automatic signal annotations were reviewed by two experts on the mapping system, and advanced signal features were extracted from bipolar and unipolar EGMs using bespoke software used in previous studies.^9,13,15,16^ A graphical user interface was used to semi-automatically review and correct annotations in MATLAB. A total of 36 VTs were mapped, 67 VT critical sites were localized, and the full diastolic pathway (including early, mid-, late diastolic) was identified in 12 VTs. The detailed VT distribution is shown in Supplementary material online, *Table S1*.

### Signal processing

Unipolar and bipolar EGMs were recorded using standard bandwidths of 0.5–500 and 30–300 Hz, respectively, with a sampling frequency of 2034.5 Hz. Data were then downloaded for offline analysis using custom algorithms implemented in MATLAB.^9^ Unipolar and bipolar EGMs were band-pass filtered between 0.5–20 and 0.5–40 Hz, respectively.

Signal features were extracted from bipolar and unipolar EGMs and categorized into four domains: functional, spatial, spectral, and time-frequency.^17,18^ Detailed descriptions of the 46 features (22 for bipolar and 26 for unipolar, with 2 general parameters and repolarization information only for unipolar EGMs) are shown in Supplementary material online, *Table S2*. Functional domain features included the following: (i) local activation time (LAT), measured by the electro-anatomical mapping system as the latest deflection in the bipolar EGMs and reviewed by an expert blinded to the study; (ii) repolarization time (RT), measured as the time of the maximum of the first derivative of the T-wave in unipolar EGMs^16^; (iii) activation recovery interval (ARI), a surrogate of local action potential duration, was defined as ARI=RT−AT^19^; (iv) EGM duration, measured by thresholding filtered and rectified signals; (v) EGM amplitude, defined as peak-to-peak amplitude of both unipolar and bipolar EGMs; (vi) number of deflections, defined as the number of negative deflections with amplitude exceeding 20% of the signal amplitude; and (vii) change rate, defined as the maximum and mean values of the absolute first derivative of the signal. Spatial domain features represent heterogeneity of activation and repolarization across the tissue and included spatial gradients of AT, RT, and ARI,^20^ with spatial gradients defined as the mean absolute change between neighbouring points within 10 mm normalized by the distance.^21^ Time-frequency domain features provide a representation of the EGMs in a joint temporal and spectral space, which allows to determination of time of occurrence, velocity, and amplitude of EGM oscillations. Time-frequency spectra were computed using a distribution from Cohen’s class (Smoothed Pseudo Wigner-Ville Distribution),^22^ with a time, frequency, and kernel shape parameters equal to 0.03, 0.15, and 0.25, respectively. The frequency domain was divided into 40 Hz spectral bands ranging from 0 to 160 Hz for bipolar EGMs and 20 Hz band ranging from 0 to 80 Hz for unipolar EGMs, and normalized power was measured for each spectral band. Features representing the spectral domain included the central signal frequency, measured as the average frequency weighted by the amplitude of the power spectral density^23^ and the number of spectral peaks.

As shown in the Supplementary material, most of functional and time-frequency features were separately measured from two signal segments: the first one included the ventricular activation period, defined as the interval within the QRS complex on the surface ECG, and the second included the post-QRS intervals. The rationale for extracting signal features from the post-QRS interval was to capture information related to both abnormal conduction (in bipolar EGMs) and repolarization (in unipolar EGMs).

To reduce the impact of using multiple substrate maps from the same pig, binary variables were created to represent the type of map (SR, LV, RV, BIV pacing) from which EGMs were extracted.

Features with skewed distributions were log-transformed, and all continuous features were normalized to have 0 mean and a standard deviation of 1.^24^

### Statistical analysis

**Figure 1:**
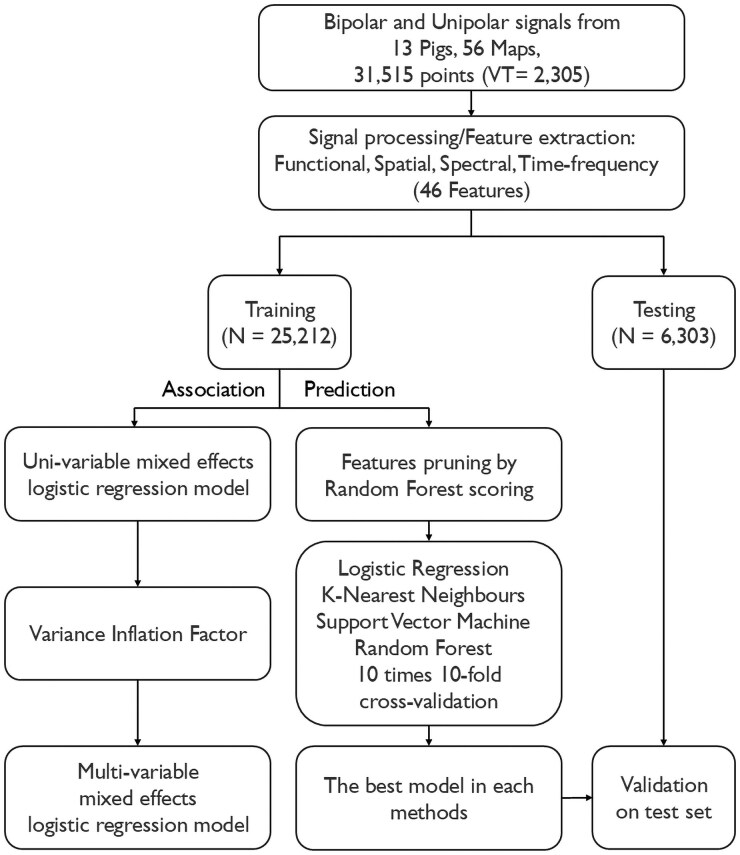
The process for data analysis.

The statistical analysis comprises two parts (*Figure 1*). Mixed-effect logistic regressions were used to determine the association between signal features and VT critical sites, while machine learning algorithms were developed to automatically predict VT critical sites. Statistical analysis was conducted by considering features extracted from each mapping point separately. Mapping points within 6 mm of a VT critical site were considered as potential ablation targets (i.e. the positive class we want to predict), and the rest were considered as non-ablation targets (i.e. the negative class). Data from all substrate maps were randomly partitioned into two groups, a large group of model development or training (80% data) and a smaller held-out group for testing (20% data). To avoid data leakage, mapping points identifying a same VT critical site were placed either within the training or test sets, but they were not allowed to be split between training and test groups.

Heatmaps were produced to assess pairwise correlations between signal features using both Pearson’s and Spearman’s correlations (Supplementary material online, *Figure S1*).^25,26^

Single-variable mixed-effects logistic regressions were used to explore the relationship between features and VT critical sites in the training set,^27,28^ using each substrate map as the random effect to reduce possible pseudo-replication. Odds ratios and 95% confidence intervals (CIs) were calculated for each feature. In the multivariable model, only signal features associated with potential ablation targets (Bonferroni corrected *P* < 0.05) were included. Collinearity was reduced by removing features with variance inflation factor above 10, where the inflation factor measures the degree of feature’s variance increase due to collinearity.^29^

### Machine learning approach

Three machine learning approaches, support vector machine (SVM), K-nearest neighbours (KNN), and random forest (RF), were trained to predict the EGMs related to VT ablation targets.^30–32^ All machine learning algorithms were executed in MATLAB 2022b. For SVM, the *fitcsvm* was used with Gaussian kernel and the box constraint parameter was adjusted to 1. In KNN, 10 nearest neighbours were counted with Euclidean distance by *fitcknn*. The RF was achieved by the *TreeBagger* function with 100 decision trees and minimum leaf size of 1.

A feature pruning method based on RF importance scoring was performed to reduce the model complexity and optimize performance.^33^ Features were sequentially added to the model for validation, from the highest to the lowest importance score. Principal component analysis with 13 components representing 90% of total variance was also implemented to compare the prediction performance (Supplementary material online, *Figure S2*). The classic 10-fold cross-validation was performed 10 times to obtain a more comprehensive assessment during training. The positive class was allocated into each fold based on VT points while the negative class was randomly selected. Stratified under-sampling strategies were adopted during the cross-validation with ratios of 1:1 and 1:5 (positive: negative class) to reduce class imbalance (1:14 in original data).

Different signal types and substrate maps were modelled using the optimal training strategies. To explore the influence of mapping and pacing strategies on ablation targets prediction, 10 distinct prediction models were developed using both unipolar and bipolar EGMs from (i) all maps, (ii) SR maps, (iii) LV pacing maps, and (iv) RV pacing maps; only using unipolar EGMs from (v) SR maps, (vi) LV pacing maps, and (vii) RV pacing maps; and only using bipolar EGMs from (viii) SR maps, (ix) LV pacing maps, and (x) RV pacing maps. The BIV maps were not separately modelled due to limited number of mapping points.

Sensitivity analysis was performed to investigate the relationship between discrimination and spatial precision, by computing area under the ROC curve (AUC) for increasing size of potential ablation targets regions. These were defined as regions including mapping points within increasing distance from the VT critical sites, from 3 to 10 mm.

To appraise the model performance, F1-score, positive predictive value (PPV), sensitivity, specificity, and AUC were calculated during 10 times 10-fold cross-validation, with the cut-off point closest to the upper left corner of the ROC curve. The model with the highest AUC across all cross-validation iterations was used to assess the model performance on the test set for each modality. Since previous studies have mainly focused on identification of ablation targets using single EP parameters, results of our integrated approach were compared with those obtained using *GradAT*, which relates to conduction velocity and isochronal crowding, *GradARI*, which relates to repolarization dispersion, and voltage, separately. The mixed-effects logistic regression model was also evaluated using the same approach as benchmark.

## Results

**Figure 2:**
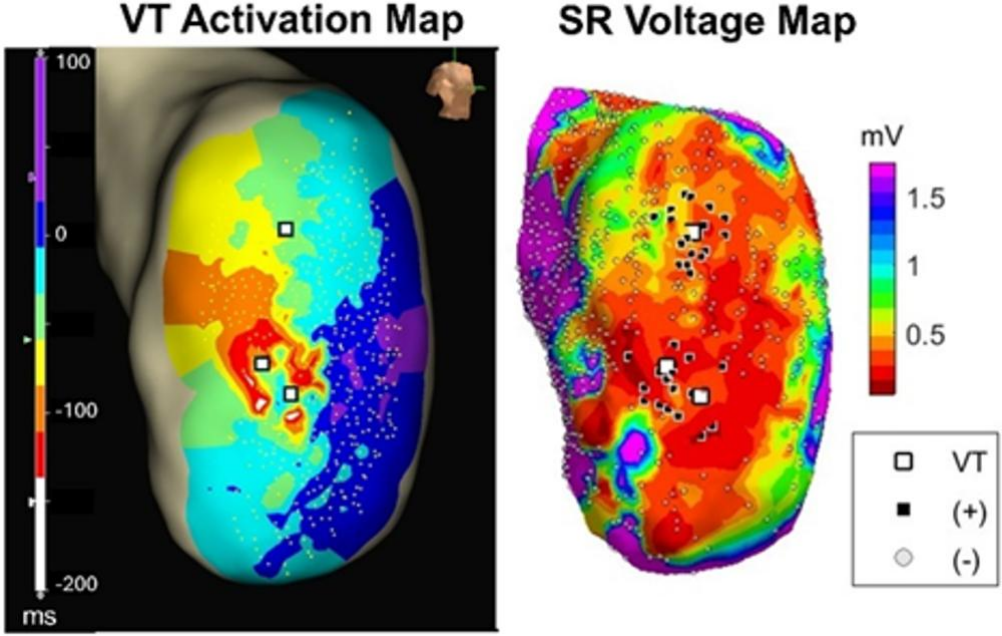
Ventricular tachycardia activation map and sinus rhythm voltage map: the left map is the activation map during ventricular tachycardia, showing a re-entrant circuit with early (bottom white squares), mid- (middle white squares), and late diastolic (top white squares) signals. The right map is the voltage map in sinus rhythm indicating a large left ventricle anterior scar. Black squares and grey circles represent sites which belong to the positive (within 6 mm from ventricular tachycardia critical sites) and negative classes, respectively.

*Figure 2* shows an example of the VT activation map and the corresponding SR voltage map. On the left, the VT activation map shows the VT critical sites (i.e. early, mid-, and late diastolic signal sites) verified by inspecting the EGMs, while on the right, superimposed to the voltage map, the black dots represent the potential ablation targets (mapping points within 6 mm from the VT critical sites, positive class) that we want to predict.

After removing duplicated signals, 31 515 bipolar and unipolar EGMs collected from different mapping points were retained for subsequent analysis. Two thousand and three hundred five (7.31%) mapping points were labelled as potential ablation targets (positive class). Characteristics of all substrate maps, including point density, are shown in *Table 1*. The map containing the highest number of EGMs was SR, with a median number of 1091 EGMs per map, followed by SE pacing in the RV and LV, with medians of 625 and 577, respectively. The map with the fewest EGMs was S1S2 from BIV, with a median of only 184 EGMs/map. Sinus rhythm also contained the highest number of potential ablation targets, with a median of 69 per map, whereas the S1S2 pacing in the LV had the fewest.

**Table 1:** Distribution of signal points and VT critical sites in each substrate maps

Substrate map	Number of pigs	Mapping points per map	VT critical sites per map
		Median (IQR)	Median (IQR)
SR	10	1091 (543–1649)	69 (51–86)
RV (SE)	10	625 (452–727)	40 (15–51)
RV (S1S2)	10	284 (157–431)	26 (9–39)
LV (SE)	10	577 (402–867)	46 (16–88)
LV (S1S2)	5	320 (226–366)	16 (1–29)
BIV (SE)	8	281 (194–469)	34 (18–49)
BIV (S1S2)	3	184 (177–283)	16 (5–59)
Total	56	460 (257–741)	32 (15–61)

**Figure 3:**
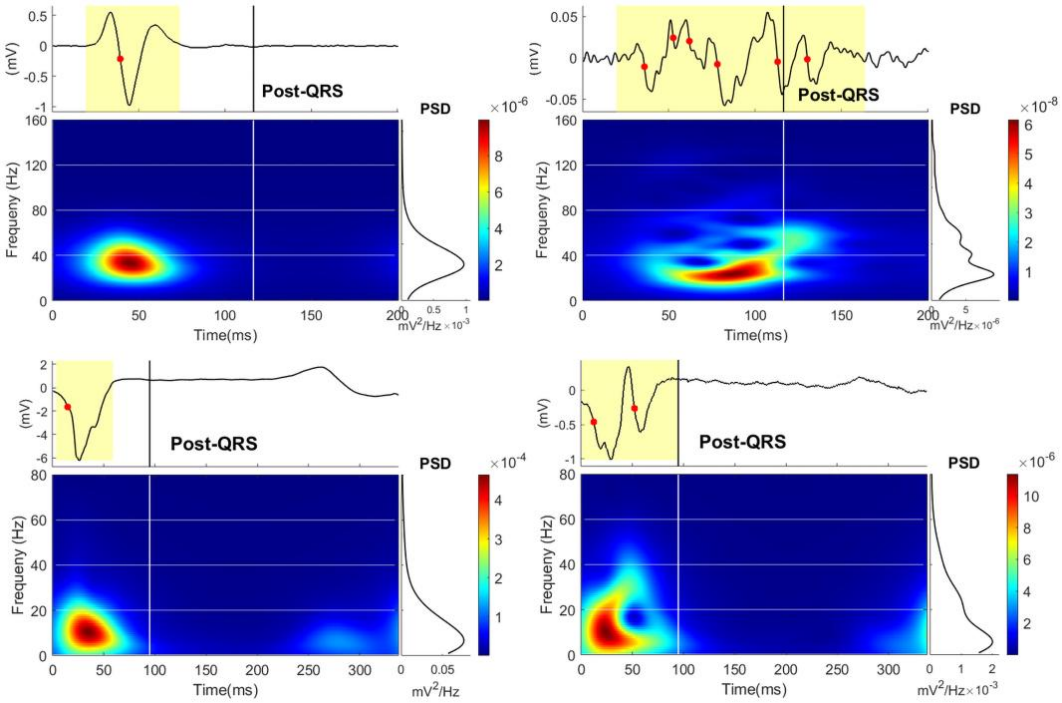
Features in bipolar and unipolar electrograms. The top and bottom panels represent bipolar and unipolar electrograms, respectively. The bipolar signals were collected from Pig 13 in sinus rhythm and the unipolar signals were collected from Pig 11 in sinus rhythm. The left and right panels represent electrograms from normal and critical ventricular tachycardia sites. The yellow region shows the signal duration (in bipolar electrograms) and the duration of the QRS complex (in unipolar electrograms), the red points represent a signal deflection, and the black vertical line is the end of the QRS complex from the body surface electrogram. The time-frequency distributions were divided into eight regions by the white cross lines for different periods and energy bands. Before, the end-point of QRS was the QRS region and after that was the post-QRS region.

*Figure 3* visualizes some of the signal features from EGMs recorded at a VT critical site compared with normal cardiac sites from both bipolar and unipolar EGMs.

### Association between signal features and critical sites

In unadjusted, single-variable mixed-effects logistical regressions, 39 of the 46 signal features investigated in this study were significantly associated with VT critical sites (*P* < 0.0011; Supplementary material online, *Table S3*). Strong correlations between features were found in pairwise correlation matrices reported as heatmaps in Supplementary material online, *Figure S1*. For instance, ARI was negatively correlated with most features. The central frequency of both bipolar and unipolar signals was positively correlated with total energy, voltage, and signal change rate but negatively correlated with duration.

In the multivariable model, 10 signal features remained significantly correlated with potential VT ablation targets (*Table 2*; Supplementary material online, *Table S4*). As expected because of the strong correlation with signal amplitude, the total energy of bipolar EGMs ($E_{B,QRS}^{0-160}$) was strongly and negatively associated with potential ablation targets, and a similar association was found for unipolar and bipolar power in lower spectral bands during ventricular activation ($R_{B,QRS}^{40-80}$ and $R_{U,QRS}^{20-40}$) and repolarization ($R_{U,T}^{20-40}$). Interestingly, elevated power in higher frequency components or during post-QRS intervals ($R_{B,QRS}^{120-160}$ and $R_{B,P}^{40-80}$) was positively associated with potential ablation targets. Furthermore, increased central frequency ($f_{U}$) and reduced rate of change ($max\left|\frac{dU_{T}}{dt}\right|$) of unipolar EGMs were also associated with potential ablation targets. Altogether, these results suggest that bipolar EGMs more likely to be associated with ablation targets have low-amplitude, high-frequency late components, while unipolar EGMs have low amplitude, low rate of change, and relatively flat T-waves. Increased spatial repolarization heterogeneity, measured by *GradARI* and *GradRT*, is also confirmed to be pro-arrhythmic.

**Table 2:** Odds ratios and *P*-values for features in multivariable logistic regression model

Feature	Definition	Odds ratio (95% CI)	*P*-value
$E_{B,QRS}^{0-160}$	Total signal energy within QRS complex (bipolar)	0.611 (0.549–0.681)	3.10E-19*
$R_{B,QRS}^{120-160}$	Fractional energy within QRS and 120–160 Hz (bipolar)	1.266 (1.181–1.358)	3.79E-11*
$R_{B,P}^{40-80}$	Fractional energy post-QRS and within 40–80 Hz (bipolar)	1.266 (1.134–1.413)	2.52E-05*
$f_{U}$	Central of mass frequency from PSD function (unipolar)	1.293 (1.129–1.479)	1.96E-04*
*GradARI*	Spatial gradients of activation recovery interval	1.158 (1.056–1.271)	1.83E-03*
$R_{U,T}^{20-40}$	Fractional energy within T-wave and 20–40 Hz (unipolar)	0.890 (0.823–0.962)	3.51E-03*
$R_{U,QRS}^{20-40}$	Fractional energy within QRS and 20–40 Hz (unipolar)	0.818 (0.71–0.942)	5.23E-03*
$R_{B,QRS}^{40-80}$	Fractional energy within QRS and 40–80 Hz (bipolar)	0.86 (0.763–0.971)	1.45E-02*
$P_{B}$	Number of peaks in PSD function (bipolar)	1.049 (1.007–1.094)	2.34E-02*
$max\left|\frac{dU_{T}}{dt}\right|$	Maximum of absolute first derivative in T-wave (unipolar)	0.831 (0.704–0.98)	2.81E-02*
*GradRT*	Spatial gradients of repolarization time	1.071 (0.989–1.16)	8.95E-02
$A_{U}^{T}$	Peak-to-peak T-wave amplitude (unipolar)	1.057 (0.981–1.139)	1.47E-01
$D_{U}$	Signal duration (unipolar)	0.959 (0.903–1.018)	1.71E-01
$A_{U}$	Peak-to-peak signal amplitude (unipolar)	0.926 (0.817–1.048)	2.24E-01
$D_{B}$	Signal duration (bipolar)	0.965 (0.905–1.029)	2.73E-01
*RT*	Repolarization time (unipolar)	1.068 (0.943–1.211)	3.00E-01
$Def_{B}$	Number of deflections within QRS (bipolar)	1.031 (0.969–1.096)	3.40E-01
$max\left|\frac{dU_{QRS}}{dt}\right|$	Max of absolute first derivative within QRS (unipolar)	0.941 (0.824–1.074)	3.65E-01
$R_{U,QRS}^{0-20}$	Fractional energy within QRS and 0–20 Hz (unipolar)	1.072 (0.892–1.288)	4.61E-01
$max\left|\frac{dU_{T}}{dt}\right|$	Max of absolute first derivative within T-wave (unipolar)	0.946 (0.811–1.104)	4.81E-01
$P_{U}$	Number of peaks in PSD function (unipolar)	0.962 (0.853–1.085)	5.24E-01
$A_{B}^{p}$	Peak-to-peak signal amplitude post-QRS (bipolar)	1.012 (0.945–1.083)	7.40E-01
$R_{U,QRS}^{60-80}$	Fractional energy within QRS and 60–80 Hz (unipolar)	0.983 (0.871–1.11)	7.86E-01
$ARI_{B}$	Activation recovery interval (bipolar): $RT-LAT_{B}$	1.012 (0.916–1.118)	8.20E-01
*GradAT*	Spatial gradients of activation time	1.008 (0.942–1.078)	8.23E-01

^*^ *P* < 0.05.

### Prediction of potential ablation targets

*Table 3* presents the importance scores for each feature calculated by RF based on training EGMs. Comparing with models trained after PCA or models that used all features, models trained using features selected based on the 20 most important features identified by RF achieved higher AUC. The 1:5 under sampling strategy showed a comparable result with those obtained without re-sampling, both higher than under sampling at 1:1 ratio (see Supplementary material online, *Table S5*).

**Table 3:** RF scoring calculated from training data including both unipolar and bipolar EGMs from all maps, from the most to the least important feature

Rank	Features	Score	Rank	Features	Score	Rank	Features	Score
1	*RT*	1.593	17	$R_{B,QRS}^{120-160}$	1.111	33	$R_{B,P}^{0-40}$	0.819
2	$D_{U}$	1.462	18	$A_{U}$	1.103	34	$R_{B,P}^{120-160}$	0.797
3	$max\left|\frac{dU_{QRS}}{dt}\right|$	1.356	19	$R_{U,T}^{40-60}$	1.074	35	$mean\left|\frac{dB_{QRS}}{dt}\right|$	0.758
4	$R_{U,T}^{0-80}$	1.337	20	$R_{U,QRS}^{40-60}$	1.050	36	$R_{B,P}^{80-120}$	0.733
5	$max\left|\frac{dU_{T}}{dt}\right|$	1.307	21	$E_{B,P}^{0-160}$	0.993	37	$R_{B,QRS}^{0-40}$	0.724
6	$A_{U}^{T}$	1.297	22	*GradRT*	0.974	38	$f_{B}$	0.715
7	$ARI_{B}$	1.297	23	$R_{B,P}^{40-80}$	0.963	39	$A_{B}^{p}$	0.705
8	$R_{U,T}^{60-80}$	1.283	24	$R_{U,QRS}^{0-20}$	0.962	40	*GradAT*	0.635
9	$LAT_{B}$	1.247	25	$R_{B,QRS}^{80-120}$	0.959	41	$mean\left|\frac{dB_{p}}{dt}\right|$	0.612
10	$R_{U,QRS}^{60-80}$	1.209	26	$R_{U,QRS}^{0-80}$	0.931	42	$P_{B}$	0.585
11	$mean\left|\frac{dU_{QRS}}{dt}\right|$	1.169	27	$E_{B,QRS}^{0-160}$	0.929	43	$max\left|\frac{dB_{p}}{dt}\right|$	0.540
12	$R_{U,T}^{0-20}$	1.148	28	$R_{B,QRS}^{40-80}$	0.923	44	$Def_{U}$	0.514
13	$A_{B}$	1.146	29	$f_{U}$	0.868	45	$Def_{B}$	0.503
14	$D_{B}$	1.146	30	$R_{U,T}^{20-40}$	0.865	46	$P_{U}$	0.470
15	$mean\left|\frac{dU_{T}}{dt}\right|$	1.142	31	$R_{U,QRS}^{20-40}$	0.858			
16	*GradARI*	1.124	32	$max\left|\frac{dB_{QRS}}{dt}\right|$	0.845			

By sequentially adding features based on RF importance score, both KNN and RF achieved stable AUC in training and test after adding the top 15 features, while the SVM reached optimal AUC after adding 20 features (see Supplementary material online, *Figure S3*). To ensure model stability and avoid signal information loss, the top 20 features selected by RF were included for training without under sampling for each model.

**Figure 4:**
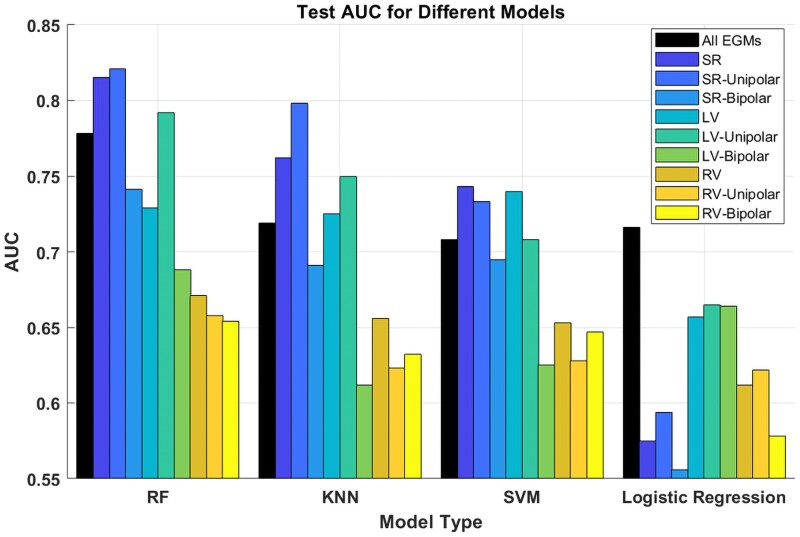
Test results for different approaches with different data set (two signal types: bipolar and unipolar; three maps: sinus rhythm, left ventricle, and right ventricle).

*Figure 4* shows the performance (AUC) of KNN, SVM, RF, and logistic regression in the held-out test set, while detailed average performance in both training and held-out test sets is shown in Supplementary material online, *Tables S11–S19*.

Across SR and pacing modalities (SR, RV, or LV) and type of signals (unipolar vs. bipolar EGMs), RF provided the best discrimination of potential ablation targets. Support vector machine and KNN showed a comparable overall performance, which was better than logistic regression’s performance. Compared with bipolar EGMs, unipolar EGMs showed superior classification performance in all models (∼0.1 advantage in test AUC). Random forest models for different configurations showed AUC with partially overlapping CIs [e.g. SR: 0.757, 95% CI (0.736–0.778), LV: 0.752 95% CI (0.745–0.777), RV: 0.739, 95% CI (0.719–0.758)]. However, when the models were evaluated in the held-out test set, the model trained on unipolar EGMs collected in SR (AUC = 0.821, sensitivity = 81.4%, specificity = 71.4%; *Table 4*) and the model trained using both unipolar and bipolar EGMs in SR (AUC = 0.815, sensitivity = 72.9%, specificity = 75.6%) were markedly superior to the rest.

**Table 4:** Training and test results for different models with the top 20 RF features on unipolar EGMs from SR map; features included in logistic were selected by VIF to remove multi-collinearity

Training	AUC	F1-score (%)	PPV (%)	Sensitivity (%)	Specificity (%)
Logistic	0.640 (0.611–0.670)	18.7 ± 6.9	11.1 ± 4.5	63.6 ± 18.1	64.8 ± 11.8
KNN	0.700 (0.680–0.720)	23.3 ± 7.9	14.9 ± 5.6	58.2 ± 18.0	78.7 ± 2.7
SVM	0.671 (0.653–0.688)	23.0 ± 8.0	14.7 ± 6.2	61.0 ± 10.9	73.9 ± 11.1
RF	0.729 (0.709–0.749)	22.3 ± 7.1	13.6 ± 5.2	67.7 ± 12.0	70.0 ± 9.7

Test	AUC	F1-score (%)	PPV (%)	Sensitivity (%)	Specificity (%)
Logistic	0.594	13.5	7.5	63.6	55.3
KNN	0.798	27.2	16.5	77.1	77.7
SVM	0.733	22.1	13.3	64.4	76.0
RF	0.821	23.9	14.0	81.4	71.4

The general model trained using all signals and maps also showed a satisfactory performance with the test AUC of 0.778 and both sensitivity and specificity of about 70% (see Supplementary material online, *Table S6*). All machine learning algorithms showed low F1-score and PPV during both training and test, which is a common finding in unbalanced data sets. The top 20 features selected by RF for each model are shown in Supplementary material online, *Table S8*.

**Figure 5:**
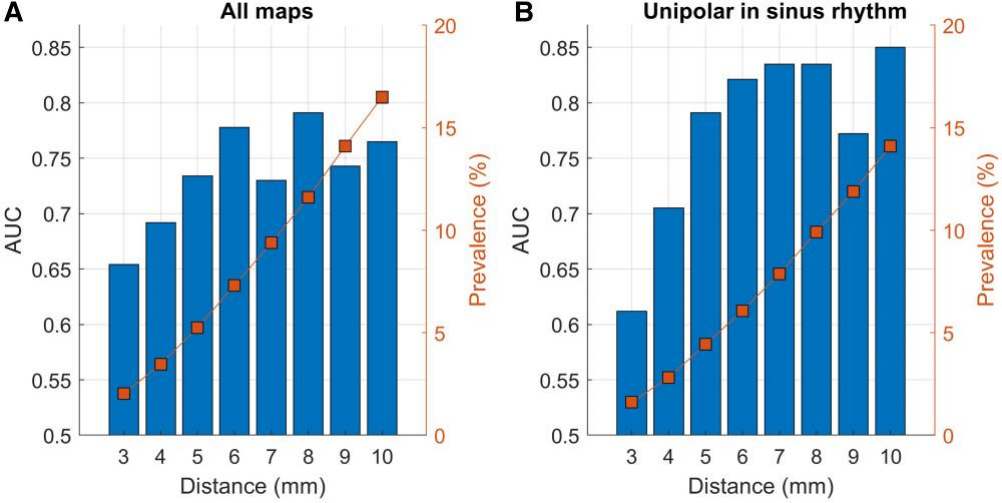
Test results for random forest algorithm with different distance settings in (*A*) including all electrograms from all maps and (*B*) including unipolar electrograms from sinus rhythm map. The blue bars represent the area under the ROC curve where the orange lines show the prevalence of positive class (potential ablation targets) at the specific distance.

In the primary analysis, potential ablation sites were defined as mapping points within 6 mm from VT critical sites. Sensitivity analyses assessed the impact of defining potential ablation sites based on increasing distance from the VT critical site. Results show that in the test set (see Supplementary material online, *Table S10*), for distances ≥ 6 mm, AUC ranged between 0.75 and 0.85, while for distances < 6 mm, AUC decreased rapidly towards 0.65 along with prevalence, which fall below 2%, for distance equal to 3 mm (*Figure 5*).

Traditional models based on single EP parameters showed reduced ability to discriminate potential ablation targets, with AUC equal to 0.58, 0.57, and 0.67 for *GradAT*, *GradARI*, and voltage, respectively (see Supplementary material online, *Table S7*).

## Discussion

The aim of this study was to develop and test machine learning models to identify potential VT ablation targets using EGM features extracted from substrate maps collected in SR or pacing. To achieve this, we developed a porcine model of chronic myocardial infarction^13^ and mapped VTs to identify critical components of the circuits. The main results are as follows: (i) RF algorithms provided the best discrimination, with AUC = 0.821 and sensitivity and specificity of 81.4% and 71.4%, respectively; (ii) models trained and tested on maps collected in SR and using unipolar EGMs demonstrate best performance; (iii) distinct signal features associated with VT ablation targets were identified for both bipolar and unipolar EGMs; and (iv) repolarization features extracted from unipolar EGMs demonstrated significant contribution in prediction of ablation targets across different models.

Despite important advances in recent years, catheter ablation for VT requires improvements in many aspects, including procedure duration and efficacy. This study demonstrates that a data-driven approach which combines advanced signal processing and machine learning may offer novel solutions. Automatic identification of potential ablation targets from substrate mapping could support clinicians during the procedure, making it safer (avoiding VT induction), shorter (avoiding extensive visual inspection of maps) and potentially more accurate.

The EGM signal features used in this study were based on state-of-the-art signal processing and provided a detailed description of signal morphology in the temporal, spectral, and time-frequency domains as well as a description of spatial functional properties. To explain possible mechanisms underpinning our results, we used multivariable logistic regressions and RF’s feature importance scoring (*Graphical Abstract*). Logistic regressions identified significant associations between ablation targets and signal features related to scar (e.g. reduced energy of bipolar EGMs within the QRS), abnormal conduction (increased high-frequency components during and after QRS), and repolarization (increased spatial gradients, lower change rates, and flatter T-waves in unipolar EGMs). Interestingly, EGMs showing larger high-frequency fractional power were not necessarily associated with late potentials, suggesting that advanced signal processing may identify more subtle arrhythmogenic signal patterns. Signal features related to scar, abnormal conduction, and repolarization were also ranked among the most important in RF models (*Table 3*; Supplementary material online, *Table S9*). Altogether, this suggests that our machine learning approach is based on features linked to well established mechanisms of re-entry.^34^ In our chronic infarct model, the scar serves as an anatomical substrate, which alters both excitation and repolarization, and as demonstrated by experimental and theoretical studies, the simultaneous combination of slow conduction and dispersion of repolarization increases vulnerability to re-entry.^8,9,34,35^

Interestingly, single-feature models showed limited accuracy (AUC < 0.67), indicating that it is challenging to identify ablation targets in substrate maps relying solely on single indices. The superiority of machine learning approaches (e.g. RF) with respect to traditional methods (e.g. regressions) can be explained by their ability to model complex, non-linear interactions between multiple parameters.

Sensitivity analysis demonstrated a trade-off between discrimination (measured as AUC) and precision (i.e. the size of the ablation target region). This is a somehow expected limitation, because reducing the size of the region defining the potential ablation targets not only increases class imbalance, but it also reduces the number of VT critical sites included in the analysis. This is because VT critical sites and potential ablation targets are identified from different maps (VT and substrate maps, respectively) with not perfectly overlapping coverage. In summary, the proposed approach can accurately localize ablation targets with a spatial resolution ≥ 6 mm. While this is a limitation, in practice, multiple ablations are usually clustered together to consolidate lesions around critical sites.

Results from different modalities showed that the model using unipolar EGMs alone in SR data provided the best discrimination (AUC = 0.821), followed by the model trained using both bipolar and unipolar EGMs collected in SR. This suggests that high-density maps collected in SR (with approximately double the number of points than pacing maps) could be more useful for the identification of potential ablation targets using signal feature-based machine learning approaches and highlights the potential usefulness of including unipolar mapping in electrophysiological studies. It is possible that machine learning approaches may perform better with larger numbers of mapping points, even if these are collected in SR as opposed to during extra-stimulation pacing aimed at unmasking pro-arrhythmic substrate. There is a trade-off between the weighting of the signal’s physiologically useful information and the number of the signals influencing the machine learning algorithm.

The higher discriminative value of signal features extracted from unipolar EGMs as compared with bipolar EGMs is also demonstrated by the fact that most of the top 20 features selected by RF scoring in the general model came from unipolar EGMs. For instance, repolarization time, which is measured from unipolar EGMs, was ranked among the top 3 features by RF scoring in 4 models and among the top 10 in all models that included unipolar EGMs. ARI, QRS duration, LAT, and high-frequency power from unipolar EGMs were also frequently ranked as top 10 features in different models (see Supplementary material online, *Table S9*). Possible explanations for the higher discriminative value of unipolar EGMs may include the contribution of repolarization-related features exclusively derived from unipolar signals and a clear and distinct morphology including information from both local and remote sources.^36,37^

It is worth noting that model performance was characterized by a relatively low F1-score and PPV in both training and test. This may be in part due to a significant class imbalance. In fact, for fixed levels of sensitivity and specificity, PPV and F1-score may decrease sharply with decreasing prevalence (i.e. smaller positive class).^38^

Compared with established approaches to substrate mapping,^7,34,39^ which usually focus on a single electrophysiological parameter, our approach was to integrate multiple electrophysiological characteristics in a single model using advanced signal processing. Advanced signal processing of EGMs has been previously implemented.^40–45^ For example, Lin *et al.*^42^ applied simultaneous amplitude frequency EGM transformation mapping to identify the conduction channels of VT. It is challenging to compare our model performance with other substrate-based methods, as most of these studies optimized or proposed a reference threshold to identify the VT critical sites and used different metrics for evaluation.^43,44^ Machine learning was previously applied to VT mapping data,^12,45^ but applications were limited to the identification of abnormal EGMs. For example, Baldazzi *et al*.^12^ achieved 93.1% accuracy in identifying abnormal ventricular potentials (39% of all signals analyzed) using multi-domain features with ensemble tree methods. However, identification of abnormal signals differs from identification of VT critical sites because the former may not be related to a VT circuit.

### Limitations

These data come from a relatively small porcine study and used endocardial mapping only. While an animal model has advantages over a human model, including extensive and detailed VT mapping would not be feasible in patients. An inherent limitation of VT mapping is that during an electrophysiological study, it is impossible to induce all possible VTs. This may translate in an underestimation of the model performance, because some false positives (mapping sites erroneously predicted to coincide with VT critical sites) could be true positive (mapping sites correctly predicted to be non-induced VT critical sites). Despite advanced signal filtering and careful re-annotation were performed to distinguish near-field and far-field signals, analysis and categorization of intracardiac EGMs in VT patients remain challenging. Another limitation is that sites with diastolic potentials were not confirmed through entrainment mapping or ablation in this study. While this limitation cannot be fully addressed with experimental data, it could be partially answered using advanced digital twins or personalized virtual-heart technology to reproduce all possible VT circuits.^46–48^ Applying our model to non-invasive mapping modalities, such as electrocardiographic imaging,^49,50^ may provide a safer and more convenient approach to identifying VT ablation targets.

This work remains hypothesis generating as in-human validation is required for clinical translation. Future work may focus on combining more advanced high-density mapping techniques with refined classification approaches with digital twins to achieve more precise localization of ablation targets.

## Conclusion

This study proposes a machine learning approach for the identification of VT ablation targets during substrate mapping in SR or pacing. In this porcine model of myocardial infarction, this strategy predicted critical components of VT circuits with good accuracy (AUC = 0.821) and identified the most relevant signal features. These data should encourage further model development in patients.

## Supplementary Material

ztaf064_Supplementary_Data

## Data Availability

The data underlying this article will be shared on reasonable request to the corresponding author.

## Lead author biography

Xuezhe Wang is a third year PhD student under supervision of Dr Michele Orini, in the Institute of Cardiovascular Science, University College London, UK. The PhD project focuses on developing advanced artificial intelligence approach to predict the ablation target for ventricular tachycardia using substrate-based information.
