# Supplementary material for: Machine learning approach for automated localization of ventricular tachycardia ablation targets from substrate maps: development and validation in a porcine model
Source: Eur Heart J Digit Health. 2025 Jun 10;6(4):645–55. doi: 10.1093/ehjdh/ztaf064 (PMC12282365; doi:10.1093/ehjdh/ztaf064)
Supplement: ztaf064_Supplementary_Data [file ztaf064_supplementary_data.docx]

Table 1 Distribution for VTs in different pigs. ED: early-diastolic, MD: mid-diastolic, LD: late-diastolic.

| **Pig** | **VTs** | **ED** | **MD** | **LD** |
| --- | --- | --- | --- | --- |
| Pig 1 | 4 | 3 | 1 | 4 |
| Pig 2 | 4 | 3 | 4 | 3 |
| Pig 3 | 1 | 1 | 1 | 1 |
| Pig 4 | 2 | 0 | 0 | 2 |
| Pig 5 | 2 | 0 | 2 | 0 |
| Pig 6 | 4 | 0 | 1 | 4 |
| Pig 7 | 4 | 4 | 5 | 3 |
| Pig 8 | 2 | 0 | 0 | 2 |
| Pig 9 | 2 | 0 | 1 | 2 |
| Pig 10 | 2 | 0 | 1 | 2 |
| Pig 11 | 2 | 1 | 1 | 2 |
| Pig 12 | 3 | 1 | 1 | 3 |
| Pig 13 | 4 | 2 | 3 | 4 |
| **Total** | 36 | 15 | 21 | 32 |

Table 2 Description and symbols for features included in the study.

| **Features** | **Explanation** | **Features** | **Explanation** |
| --- | --- | --- | --- |
| ${LAT}_{B}$ | Local activation time from bipolar EGMs | ${LAT}_{U}$ | Local activation time from unipolar EGMs |
| ${ARI}_{B}$ | Activation recovery index for bipolar: $RT-{LAT}_{B}$ | ${ARI}_{U}$ | Activation recovery index for unipolar: $RT-{LAT}_{U}$ |
| $RT$ | Repolarization time from unipolar EGMs | $GradRT$ | Gradients of $RT$ (in ms/mm) |
| $GradAT$ | Gradients of AT (in ms/mm) | $GradARI$ | Gradients of $ARI$ (in ms/mm) |
| $P_{B}$ | Number of peaks from PSD (Over 20% of max) of bipolar EGMs | $P_{U}$ | Number of peaks from PSD (Over 20% of max) of unipolar EGMs |
| ${Def}_{B}$ | Number of deflections within QRS of bipolar EGMs | ${Def}_{U}$ | Number of deflections within QRS of unipolar EGMs |
| $A_{B}$ | Peak to peak voltage for Bipolar EGMs (mV) | $A_{U}$ | Peak to peak voltage for unipolar EGMs (mV) |
| $A_{B}^{p}$ | The amplitude of bipolar signal in post-QRS segment | $A_{U}^{T}$ | The amplitude of unipolar signal in T-wave |
| $max\vert\frac{dB_{QRS}}{dt}\vert$ | Maximum of the absolute first derivative in QRS complex of bipolar EGMs | $max\vert\frac{dU_{QRS}}{dt}\vert$ | Maximum of the absolute first derivative in QRS complex of unipolar EGMs |
| $mean\vert\frac{dB_{QRS}}{dt}\vert$ | Mean of the absolute first derivative in QRS complex of bipolar EGMs | $mean\vert\frac{dU_{QRS}}{dt}\vert$ | Mean of the absolute first derivative in QRS complex of unipolar EGMs |
| $max\vert\frac{dB_{p}}{dt}\vert$ | Maximum of the absolute first derivative in post-QRS for bipolar EGMs | $max\vert\frac{dU_{T}}{dt}\vert$ | Maximum of the absolute first derivative in T-wave of unipolar EGMs |
| $mean\vert\frac{dB_{p}}{dt}\vert$ | Mean of the absolute first derivative in post-QRS for bipolar EGMs | $mean\vert\frac{dU_{T}}{dt}\vert$ | Mean of the absolute first derivative in T-wave of unipolar EGMs |
| $D_{B}$ | Duration time for QRS in bipolar EGMs (in ms) | $D_{U}$ | Duration time for QRS in unipolar EGMs (in ms) |
| $f_{B}$ | Central of mass frequency from the PSD for bipolar EGMs | $f_{U}$ | Central of mass frequency from the PSD for unipolar EGMs |
| $R_{B,QRS}^{0-40}$ | Fractional energy between 0-40 Hz in QRS for bipolar EGMs | $R_{U,QRS}^{0-20}$ | Fractional energy between 0-20 Hz in QRS for unipolar EGMs |
| $R_{B,QRS}^{40-80}$ | Fractional energy between 40-80 Hz in QRS for bipolar EGMs | $R_{U,QRS}^{20-40}$ | Fractional energy between 20-40 Hz in QRS for unipolar EGMs |
| $R_{B,QRS}^{80-120}$ | Fractional energy between 80-120 Hz in QRS for bipolar EGMs | $R_{U,QRS}^{40-60}$ | Fractional energy between 40-60 Hz in QRS for unipolar EGMs |
| $R_{B,QRS}^{120-160}$ | Fractional energy between 120-160 Hz in QRS for bipolar EGMs | $R_{U,QRS}^{60-80}$ | Fractional energy between 60-80 Hz in QRS for unipolar EGMs |
| $E_{B,QRS}^{0-160}$ | Total energy between 0-160 Hz in QRS for bipolar EGMs | $E_{U,QRS}^{0-80}$ | Total energy between 0-80 Hz in QRS for unipolar EGMs |
| $R_{B,P}^{0-40}$ | Fractional energy between 0-40 Hz in post-QRS for bipolar EGMs | $R_{U,T}^{0-20}$ | Fractional energy between 0-20 Hz in T-wave for unipolar EGMs |
| $R_{B,P}^{40-80}$ | Fractional energy between 40-80 Hz in post-QRS for bipolar EGMs | $R_{U,T}^{20-40}$ | Fractional energy between 20-40 Hz in T-wave for unipolar EGMs |
| $R_{B,P}^{80-120}$ | Fractional energy between 80-120 Hz in post-QRS for bipolar EGMs | $R_{U,T}^{40-60}$ | Fractional energy between 40-60 Hz in T-wave for unipolar EGMs |
| $R_{B,P}^{120-160}$ | Fractional energy between 120-160 Hz in post-QRS for bipolar EGMs | $R_{U,T}^{60-80}$ | Fractional energy between 60-80 Hz in T-wave for unipolar EGMs |
| $E_{B,P}^{0-160}$ | Total energy between 0-160 Hz in post-QRS for bipolar EGMs | $E_{U,T}^{0-80}$ | Total energy between 0-80 Hz in T-wave for unipolar EGMs |


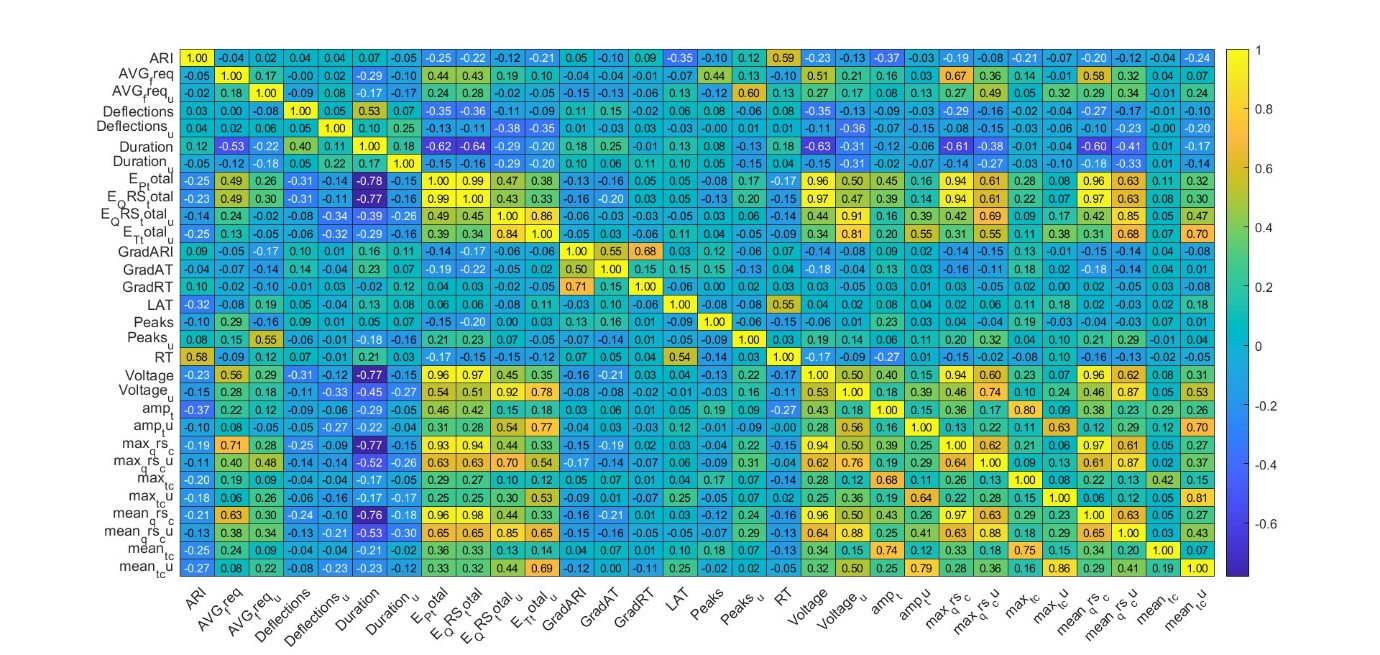


Figure 1 Pearson’s correlation matrix (upper triangular part) and Spearman’s rank correlation matrix (lower triangular part), the colour represents the correlation coefficient, from -0.6 (blue) to 1 (yellow).


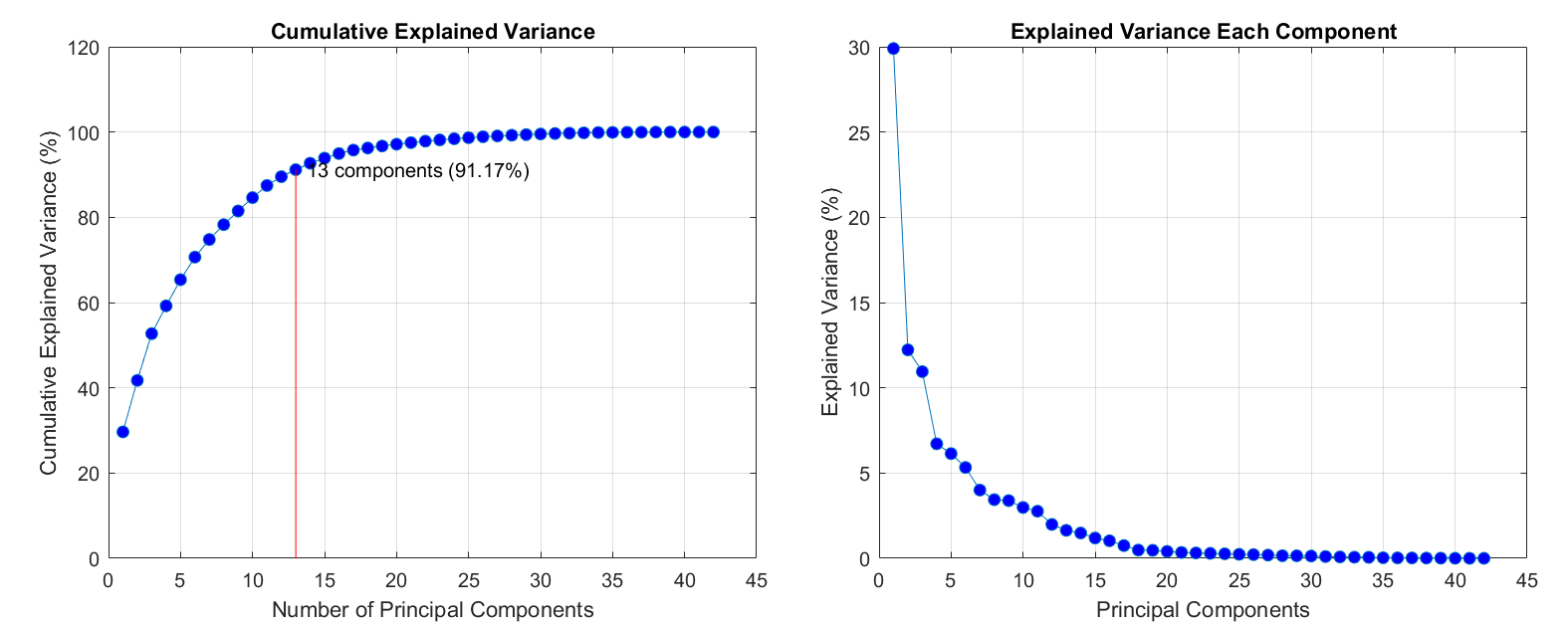


Figure 2 Cumulative explained variance in PCA, top 13 components contain over 90% of variance.

Table 3 Model results for single-variable logistic regression, *P-value<0.0011.

| **Features** | **Odd Ratios** | **95%CI (lower)** | **95%CI (upper)** | **P-value** |
| --- | --- | --- | --- | --- |
| $E_{B,QRS}^{0-160}$ | 0.55 | 0.51 | 0.58 | 5.34E-93* |
| $E_{B,P}^{0-160}$ | 0.56 | 0.53 | 0.60 | 5.19E-82* |
| $A_{B}$ | 0.58 | 0.54 | 0.61 | 1.97E-78* |
| $mean\vert\frac{dB_{QRS}}{dt}\vert$ | 0.59 | 0.55 | 0.62 | 3.49E-69* |
| $max\vert\frac{dB_{QRS}}{dt}\vert$ | 0.63 | 0.60 | 0.67 | 1.21E-56* |
| $mean\vert\frac{dU_{QRS}}{dt}\vert$ | 0.62 | 0.58 | 0.65 | 2.02E-54* |
| $max\vert\frac{dU_{QRS}}{dt}\vert$ | 0.60 | 0.56 | 0.64 | 5.53E-54* |
| $D_{B}$ | 1.35 | 1.29 | 1.40 | 6.00E-48* |
| $A_{U}$ | 0.65 | 0.62 | 0.69 | 5.37E-43* |
| $mean\vert\frac{dU_{T}}{dt}\vert$ | 0.65 | 0.61 | 0.70 | 2.32E-34* |
| $GradARI$ | 1.37 | 1.30 | 1.44 | 3.54E-33* |
| $E_{U,QRS}^{0-80}$ | 0.70 | 0.66 | 0.75 | 5.27E-32* |
| $R_{U,QRS}^{20-40}$ | 0.70 | 0.66 | 0.74 | 1.02E-31* |
| $P_{B}$ | 1.18 | 1.15 | 1.22 | 2.67E-29* |
| $E_{U,T}^{0-80}$ | 0.70 | 0.66 | 0.75 | 5.86E-27* |
| ${Def}_{B}$ | 1.30 | 1.24 | 1.36 | 9.21E-27* |
| $R_{U,QRS}^{0-20}$ | 1.48 | 1.38 | 1.60 | 5.33E-25* |
| $GradAT$ | 1.28 | 1.22 | 1.35 | 9.58E-24* |
| $R_{U,QRS}^{40-60}$ | 0.75 | 0.70 | 0.79 | 2.63E-20* |
| $max\vert\frac{dU_{T}}{dt}\vert$ | 0.70 | 0.65 | 0.75 | 2.08E-19* |
| $f_{U}$ | 0.75 | 0.71 | 0.80 | 6.22E-19* |
| $R_{U,QRS}^{60-80}$ | 0.79 | 0.75 | 0.84 | 1.65E-13* |
| $R_{U,T}^{20-40}$ | 0.82 | 0.78 | 0.87 | 1.23E-11* |
| $R_{U,T}^{0-20}$ | 1.22 | 1.15 | 1.29 | 1.09E-10* |
| $P_{U}$ | 0.73 | 0.67 | 0.81 | 2.36E-10* |
| $R_{B,QRS}^{40-80}$ | 0.86 | 0.82 | 0.90 | 3.38E-09* |
| ${ARI}_{B}$ | 1.24 | 1.15 | 1.33 | 4.99E-09* |
| $R_{U,T}^{40-60}$ | 0.86 | 0.81 | 0.91 | 8.67E-08* |
| $R_{U,T}^{60-80}$ | 0.86 | 0.81 | 0.91 | 2.81E-07* |
| $GradRT$ | 1.16 | 1.09 | 1.22 | 3.35E-07* |
| $R_{U,QRS}^{120-160}$ | 1.11 | 1.06 | 1.17 | 1.69E-05* |
| $A_{U}^{T}$ | 0.84 | 0.78 | 0.91 | 2.56E-05* |
| $R_{B,P}^{40-80}$ | 0.90 | 0.86 | 0.95 | 3.54E-05* |
| $D_{U}$ | 1.12 | 1.06 | 1.18 | 3.55E-05* |
| $A_{B}^{p}$ | 0.89 | 0.84 | 0.94 | 1.09E-04* |
| $RT$ | 1.17 | 1.08 | 1.27 | 1.59E-04* |
| $R_{B,QRS}^{0-40}$ | 1.10 | 1.05 | 1.16 | 2.04E-04* |
| ${LAT}_{B}$ | 0.86 | 0.77 | 0.94 | 1.85E-03 |
| $R_{B,P}^{0-40}$ | 1.07 | 1.02 | 1.13 | 9.26E-03 |
| $R_{B,P}^{80-120}$ | 0.94 | 0.89 | 0.99 | 1.64E-02 |
| $D_{U}$ | 1.08 | 0.99 | 1.19 | 9.91E-02 |
| $R_{B,P}^{120-160}$ | 1.03 | 0.98 | 1.08 | 3.18E-01 |
| $mean\vert\frac{dB_{p}}{dt}\vert$ | 0.88 | 0.66 | 1.16 | 3.60E-01 |
| $R_{B,QRS}^{80-120}$ | 1.01 | 0.96 | 1.06 | 6.89E-01 |
| $f_{B}$ | 0.99 | 0.94 | 1.04 | 7.75E-01 |
| $max\vert\frac{dB_{p}}{dt}\vert$ | 1.00 | 0.95 | 1.05 | 9.88E-01 |

Table 4 VIF results for the 25 features included in the multi-feature logistic regression model.

| **Features** | **VIF** | **P-value** | **Features** | **VIF** | **P-value** |
| --- | --- | --- | --- | --- | --- |
| $D_{U}$ | 1.218 | 3.55E-05 | $GradARI$ | 3.159 | 3.54E-33 |
| ${Def}_{B}$ | 1.514 | 9.21E-27 | $A_{U}$ | 3.959 | 5.37E-43 |
| $A_{B}^{p}$ | 1.662 | 0.000109 | $E_{B,QRS}^{0-160}$ | 4.094 | 5.34E-93 |
| $P_{B}$ | 1.681 | 2.67E-29 | $R_{U,QRS}^{60-80}$ | 4.394 | 1.65E-13 |
| $GradAT$ | 1.752 | 9.58E-24 | $max\vert\frac{dU_{QRS}}{dt}\vert$ | 4.538 | 5.53E-54 |
| $RT$ | 1.87 | 0.000159 | $max\vert\frac{dU_{T}}{dt}\vert$ | 5.062 | 2.08E-19 |
| $R_{B,QRS}^{120-160}$ | 1.906 | 1.69E-05 | $R_{U,QRS}^{20-40}$ | 5.884 | 1.02E-31 |
| ${ARI}_{B}$ | 2.025 | 4.99E-09 | $R_{B,P}^{40-80}$ | 5.965 | 3.54E-05 |
| $P_{U}$ | 2.039 | 2.36E-10 | $f_{U}$ | 6.402 | 6.22E-19 |
| $R_{U,T}^{20-40}$ | 2.154 | 1.23E-11 | $mean\vert\frac{dU_{T}}{dt}\vert$ | 6.929 | 2.32E-34 |
| $A_{U}^{T}$ | 2.218 | 2.56E-05 | $R_{B,QRS}^{40-80}$ | 7.158 | 3.38E-09 |
| $GradRT$ | 2.292 | 3.35E-07 | $R_{U,QRS}^{0-20}$ | 7.722 | 5.33E-25 |
| $D_{B}$ | 2.324 | 6.00E-48 |  |  |  |

Table 5 Test AUC for 3 machine learning algorithms cross different strategies using all EGMs and maps.

| **Test AUC** | **Top 20 RF Features** | | | **PCA** | | |
| --- | --- | --- | --- | --- | --- | --- |
|  | *Original* | *1:1* | *1:5* | *Original* | *1:1* | *1:5* |
| **SVM** | 0.708 | 0.695 | 0.711 | 0.653 | 0.626 | 0.650 |
| **KNN** | 0.719 | 0.703 | 0.714 | 0.606 | 0.619 | 0.624 |
| **RF** | 0.778 | 0.748 | 0.765 | 0.710 | 0.725 | 0.731 |


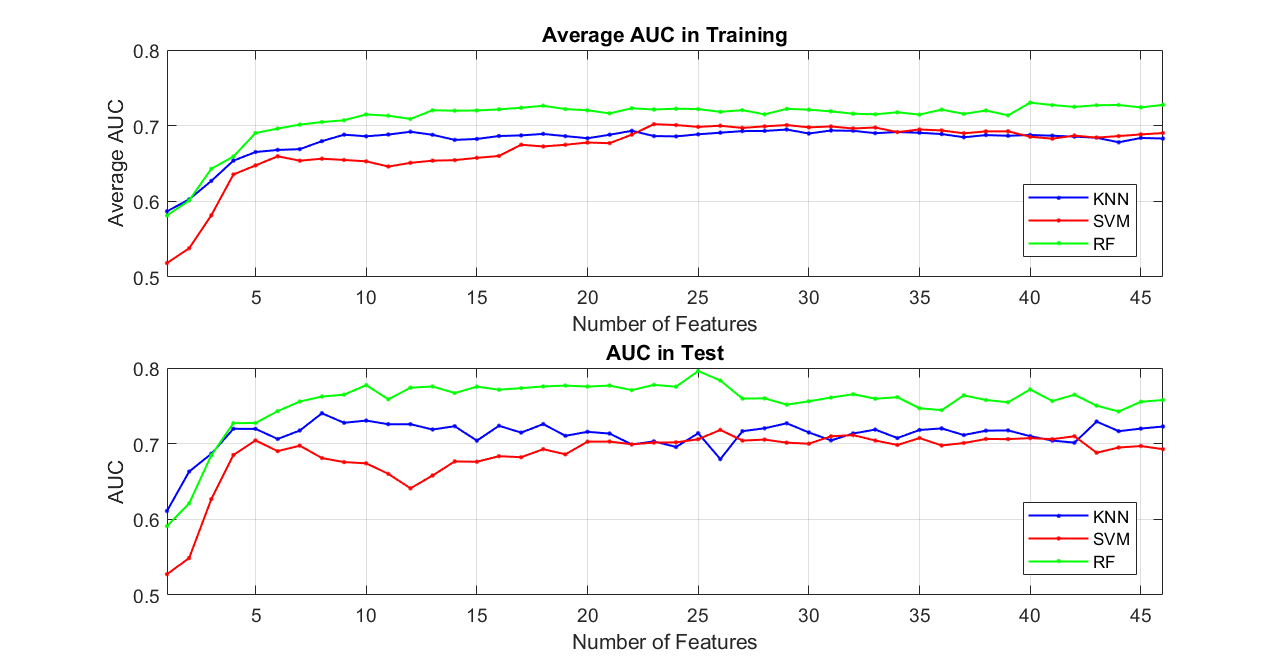


Figure 3 Average training and test AUC for 3 machine learning algorithms with adding different number of features on all EGMs and maps.

Table 6 Training and test results for different algorithms with all EGMs and maps.

| **Training (%)** | **AUC** | **Accuracy** | **F1-score** | **PPV** | **Sensitivity** | **Specificity** | |
| --- | --- | --- | --- | --- | --- | --- | --- |
| Logistic | 0.644  [0.632-0.656] | 63.7±6.5 | 19.5±4.0 | 11.7±2.7 | 59.1±8.0 | 64.1±6.9 | |
| KNN | 0.692  [0.683-0.701] | 71.4±0.9 | 23.8±3.8 | 14.9±2.7 | 61.4±8.2 | 72.3±0.1 | |
| SVM | 0.679  [0.668-0.688] | 68.4±5.2 | 22.4±4.4 | 13.9±3.3 | 61.2±5.2 | 69.0±5.6 | |
| RF | 0.729  [0.719-0.739] | 68.0±4.4 | 23.8±3.9 | 14.5±2.8 | 67.9±5.6 | 68.0±4.7 | |
| **Test (%)** | **AUC** | **Accuracy** | **F1-score** | **PPV** | **Sensitivity** | **Specificity** |  |
| Logistic | 0.686 | 65.3 | 20.0 | 12.0 | 61.2 | 65.6 |  |
| KNN | 0.719 | 71.6 | 25.0 | 15.4 | 66.6 | 72.0 |  |
| SVM | 0.708 | 68.9 | 22.5 | 13.7 | 63.7 | 69.3 |  |
| RF | 0.778 | 70.0 | 24.9 | 15.1 | 70.2 | 70.0 |  |

Table 7 The AUC for modelling with single feature ($GradAT$, $GradARI$ and Voltage) in different algorithms using all EGMs.

| **Training** | $GradAT$ | $GradARI$ | $A_{U}$ | $A_{B}$ |
| --- | --- | --- | --- | --- |
| Logistic | 0.599  [0.585-0.612] | 0.605  [0.596-0.615] | 0.584  [0.568-0.599] | 0.635  [0.622-0.647] |
| KNN | 0.520  [0.515-0.524] | 0.518  [0.514-0.523] | 0.538  [0.533-0.543] | 0.575  [0.568-0.583] |
| SVM | 0.508  [0.496-0.521] | 0.516  [0.504-0.527] | 0.507  [0.491-0.523] | 0.487  [0.472-0.503] |
| RF | 0.509  [0.505-0.512] | 0.513  [0.510-0.517] | 0.532  [0.527-0.536] | 0.565  [0.559-0.571] |
| **Test** | $GradAT$ | $GradARI$ | $A_{U}$ | $A_{B}$ |
| Logistic | 0.577 | 0.565 | 0.572 | 0.666 |
| KNN | 0.531 | 0.524 | 0.533 | 0.606 |
| SVM | 0.537 | 0.530 | 0.603 | 0.590 |
| RF | 0.540 | 0.521 | 0.512 | 0.586 |

Table 8 Top 20 features selected by RF in each model. The same colour representing the frequent top-ranked features.

| **SR** | **SR-BI** | **SR-UNI** | **LV** | **LV-BI** | **LV-UNI** | **RV** | **RV-BI** | **RV-UNI** |
| --- | --- | --- | --- | --- | --- | --- | --- | --- |
| $ARI$ | $A_{B}$ | $R_{U,QRS}^{60-80}$ | $max\vert\frac{dU_{QRS}}{dt}\vert$ | $mean\vert\frac{dB_{QRS}}{dt}\vert$ | $GradRT$ | $RT$ | $D_{B}$ | $RT$ |
| $RT$ | $R_{B,QRS}^{80-120}$ | ${LAT}_{U}$ | $E_{U,QRS}^{0-80}$ | $R_{B,P}^{80-120}$ | ${LAT}_{U}$ | $D_{U}$ | $R_{B,P}^{120-160}$ | $D_{U}$ |
| $D_{U}$ | $R_{B,P}^{0-40}$ | $D_{U}$ | $LAT$ | $R_{B,QRS}^{80-120}$ | $RT$ | $A_{U}$ | $R_{B,QRS}^{80-120}$ | ${ARI}_{U}$ |
| $R_{U,QRS}^{60-80}$ | $R_{B,P}^{80-120}$ | $GradARI$ | $A_{U}$ | $R_{B,P}^{0-40}$ | $R_{U,QRS}^{40-60}$ | $E_{U,T}^{0-80}$ | $R_{B,P}^{80-120}$ | $E_{U,T}^{0-80}$ |
| $R_{U,QRS}^{20-40}$ | $D_{B}$ | ${ARI}_{U}$ | $mean\vert\frac{dU_{QRS}}{dt}\vert$ | $R_{B,QRS}^{120-160}$ | ${ARI}_{U}$ | $mean\vert\frac{dU_{QRS}}{dt}\vert$ | $A_{B}$ | $max\vert\frac{dU_{QRS}}{dt}\vert$ |
| $max\vert\frac{dU_{QRS}}{dt}\vert$ | $LAT$ | $GradRT$ | $GradRT$ | $A_{B}$ | $A_{U}$ | $A_{U}^{T}$ | $R_{B,QRS}^{120-160}$ | $max\vert\frac{dU_{T}}{dt}\vert$ |
| $GradRT$ | $R_{B,QRS}^{120-160}$ | $A_{U}^{T}$ | $RT$ | $R_{B,P}^{120-160}$ | $R_{U,QRS}^{60-80}$ | $E_{U,QRS}^{0-80}$ | $R_{B,P}^{40-80}$ | $R_{U,QRS}^{60-80}$ |
| $E_{U,T}^{0-80}$ | $f_{B}$ | $E_{U,T}^{0-80}$ | $R_{U,QRS}^{40-60}$ | $R_{B,P}^{40-80}$ | $D_{U}$ | $mean\vert\frac{dU_{T}}{dt}\vert$ | $E_{B,QRS}^{0-160}$ | $f_{U}$ |
| $A_{U}$ | $R_{B,P}^{40-80}$ | $RT$ | $R_{U,QRS}^{60-80}$ | $E_{B,QRS}^{0-160}$ | $A_{U}^{T}$ | $ARI$ | $R_{B.P}^{0-40}$ | $mean\vert\frac{dU_{T}}{dt}\vert$ |
| $max\vert\frac{dU_{T}}{dt}\vert$ | $E_{B,P}^{0-160}$ | $max\vert\frac{dU_{T}}{dt}\vert$ | $R_{U,QRS}^{0-20}$ | $LAT$ | $R_{U,T}^{60-80}$ | $LAT$ | $f_{B}$ | $mean\vert\frac{dU_{QRS}}{dt}\vert$ |
| $A_{U}^{T}$ | $R_{B,QRS}^{40-80}$ | $R_{U,T}^{40-60}$ | $A_{B}$ | $max\vert\frac{dB_{QRS}}{dt}\vert$ | $max\vert\frac{dU_{T}}{dt}\vert$ | $R_{U,QRS}^{60-80}$ | $E_{B,P}^{0-160}$ | $GradRT$ |
| $LAT$ | $mean\vert\frac{dB_{QRS}}{dt}\vert$ | $R_{U,T}^{20-40}$ | $f_{U}$ | $A_{B}^{P}$ | $f_{U}$ | $max\vert\frac{dU_{QRS}}{dt}\vert$ | $mean\vert\frac{dB_{QRS}}{dt}\vert$ | ${LAT}_{U}$ |
| $R_{U,T}^{20-40}$ | $R_{B,P}^{120-160}$ | $GradAT$ | $mean\vert\frac{dB_{QRS}}{dt}\vert$ | $R_{B,QRS}^{40-80}$ | $R_{U,QRS}^{0-20}$ | $R_{U,QRS}^{20-40}$ | $R_{B,QRS}^{0-40}$ | $A_{U}$ |
| $E_{U,QRS}^{0-80}$ | $E_{B,QRS}^{0-160}$ | $mean\vert\frac{dU_{T}}{dt}\vert$ | $E_{B,QRS}^{0-160}$ | $E_{B,P}^{0-160}$ | $R_{U,QRS}^{20-40}$ | $R_{B,P}^{0-40}$ | $P_{B}$ | $A_{U}^{T}$ |
| $GradARI$ | $R_{B,QRS}^{0-40}$ | $R_{U,T}^{60-80}$ | $D_{U}$ | $D_{B}$ | $mean\vert\frac{dU_{QRS}}{dt}\vert$ | $max\vert\frac{dU_{T}}{dt}\vert$ | $max\vert\frac{dB_{QRS}}{dt}\vert$ | $R_{U,QRS}^{20-40}$ |
| $mean\vert\frac{dU_{QRS}}{dt}\vert$ | $max\vert\frac{dB_{QRS}}{dt}\vert$ | $A_{U}$ | $E_{U,T}^{0-80}$ | $mean\vert\frac{dB_{P}}{dt}\vert$ | $E_{U,QRS}^{0-80}$ | $R_{U,QRS}^{40-60}$ | $A_{B}^{P}$ | $R_{U,QRS}^{40-60}$ |
| $R_{U,T}^{40-60}$ | $GradAT$ | $f_{U}$ | $R_{B,QRS}^{40-80}$ | $f_{B}$ | $max\vert\frac{dU_{QRS}}{dt}\vert$ | $R_{B,QRS}^{80-120}$ | $max\vert\frac{dB_{P}}{dt}\vert$ | $GradARI$ |
| $E_{B,QRS}^{0-160}$ | $P_{B}$ | $R_{U,QRS}^{60-80}$ | $A_{B}^{p}$ | $R_{B,QRS}^{0-40}$ | $R_{U,T}^{20-40}$ | $GradRT$ | $mean\vert\frac{dB_{P}}{dt}\vert$ | $R_{U,QRS}^{0-20}$ |
| $f_{U}$ | $mean\vert\frac{dB_{P}}{dt}\vert$ | $mean\vert\frac{dU_{QRS}}{dt}\vert$ | $R_{U,QRS}^{20-40}$ | $max\vert\frac{dB_{P}}{dt}\vert$ | $E_{U,T}^{0-80}$ | $E_{B,QRS}^{0-160}$ | $R_{B.QRS}^{40-80}$ | $GradAT$ |
| $mean\vert\frac{dU_{T}}{dt}\vert$ | $A_{B}^{p}$ | $R_{U,QRS}^{40-60}$ | $R_{B,QRS}^{120-160}$ | $P_{B}$ | $GradAT$ | $f_{U}$ | $LAT$ | $E_{U,QRS}^{0-80}$ |

Table 9 Features ranking in RF scoring (top 10) across different models, and total appear time counts.

| **Features** | **Counts** | **All EGMs** | **SR** | **SR-BI** | **SR-UNI** | **LV** | **LV-BI** | **LV-UNI** | **RV** | **RV-BI** | **RV-UNI** |
| --- | --- | --- | --- | --- | --- | --- | --- | --- | --- | --- | --- |
| High-frequency energy in QRS | 9 |  | #4 | #2 | #1 | #9 | #3 | #7 | #7 | #3 | #7 |
| High-frequency energy in post-QRS | 9 | #6 | #8 | #4 | #8 |  | #2 | #10 | #4 | #2 | #4 |
| Duration | 8 | #8 | #3 | #5 |  |  | #9 | #8 | #2 | #1 | #2 |
| Voltage | 8 | #9 | #9 | #1 |  | #4 | #6 | #6 | #3 | #5 |  |
| $LAT$ | 7 | #5 |  | #6 | #2 | #3 | #10 | #2 | #10 |  |  |
| $RT$ | 7 | #1 | #2 |  | #9 | #7 |  | #3 | #1 |  | #1 |
| $ARI$ | 7 | #7 | #1 |  | #4 |  |  | #5 | #9 |  | #3 |
| Chang rate (QRS) | 5 |  | #6 |  |  | #1 | #1 |  | #5 |  | #5 |
| Voltage (post-QRS) | 5 | #4 |  |  | #7 |  | #6 | #9 | #6 |  |  |
| $GradRT$ | 4 |  | #7 |  | #6 | #6 |  | #1 |  |  |  |

Table 10 Test AUC for Random Forest algorithm with different distance settings in all EGMs (31,515) and unipolar EGMs from Sinus Rhythm map (10,959). The distance is in millimetre (mm) and followed with the prevalence of positive class (potential ablation targets).

| **All EGMs** | | **Unipolar in Sinus Rhythm** | |
| --- | --- | --- | --- |
| Distance (Prevalence%) | Test AUC | Distance (Prevalence%) | Test AUC |
| 3mm (2.02%) | 0.654 | 3mm (1.62%) | 0.612 |
| 4mm (3.47%) | 0.692 | 4mm (2.82%) | 0.705 |
| 5mm (5.25%) | 0.734 | 5mm (4.43%) | 0.791 |
| 6mm (7.31%) | 0.778 | 6mm (6.08%) | 0.821 |
| 7mm (9.39%) | 0.730 | 7mm (7.88%) | 0.835 |
| 8mm (11.6%) | 0.791 | 8mm (9.91%) | 0.835 |
| 9mm (14.1%) | 0.743 | 9mm (11.9%) | 0.772 |
| 10mm (16.5%) | 0.765 | 10mm (14.1%) | 0.850 |

Table 11 Training and test results for sinus rhythm with all EGMs.

| **Training (%)** | **AUC** | **Accuracy** | **F1-score** | **PPV** | **Sensitivity** | **Specificity** | |
| --- | --- | --- | --- | --- | --- | --- | --- |
| Logistic | 0.682  [0.651-0.712] | 69.8±11.6 | 21.1±7.4 | 12.9±5.0 | 64.7±17.3 | 70.2±12.4 | |
| KNN | 0.688  [0.669-0.708] | 76.6±2.4 | 22.2±7.0 | 14.1±4.8 | 56.5±17.8 | 78.0±2.2 | |
| SVM | 0.681  [0.663-0.698] | 73.5±10.0 | 23.7±7.8 | 15.2±6.4 | 61.7±9.5 | 74.2±10.5 | |
| RF | 0.757  [0.736-0.778] | 70.4±1.1 | 23.8±7.2 | 14.5±5.2 | 71.9±10.9 | 70.3±10.5 | |
| **Test (%)** | **AUC** | **Accuracy** | **F1-score** | **PPV** | **Sensitivity** | **Specificity** |  |
| Logistic | 0.575 | 59.0 | 12.0 | 6.8 | 51.7 | 59.4 |  |
| KNN | 0.762 | 76.2 | 24.9 | 15.0 | 72.9 | 76.3 |  |
| SVM | 0.743 | 76.6 | 23.0 | 14.0 | 64.4 | 77.3 |  |
| RF | 0.815 | 75.4 | 24.3 | 14.6 | 72.9 | 75.6 |  |

Table 12 Training and test results for sinus rhythm with only bipolar EGMs.

| **Training (%)** | **AUC** | **Accuracy** | **F1-score** | **PPV** | **Sensitivity** | **Specificity** |  |
| --- | --- | --- | --- | --- | --- | --- | --- |
| Logistic | 0.694  [0.674-0.715] | 65.6±9.8 | 20.0±5.5 | 11.8±3.7 | 68.7±11.3 | 65.4±10.4 |  |
| KNN | 0.632  [0.615-0.649] | 66.2±2.6 | 16.2±3.8 | 9.7±2.5 | 56.3±15.4 | 67.0±2.9 |  |
| SVM | 0.657  [0.639-0.675] | 66.6±7.6 | 18.9±5.6 | 11.3±3.7 | 63.0±11.3 | 66.8±8.0 |  |
| RF | 0.680  [0.659-0.702] | 66.8±9.1 | 19.6±5.7 | 11.7±3.8 | 63.8±11.9 | 67.0±9.5 |  |
| **Test (%)** | **AUC** | **Accuracy** | **F1-score** | **PPV** | **Sensitivity** | **Specificity** | |
| Logistic | 0.587 | 68.3 | 12.6 | 7.4 | 42.4 | 69.7 | |
| KNN | 0.641 | 65.0 | 15.9 | 9.1 | 61.0 | 65.2 | |
| SVM | 0.634 | 71.2 | 16.3 | 9.7 | 51.7 | 72.4 | |
| RF | 0.647 | 60.8 | 14.8 | 8.4 | 62.7 | 60.7 | |

Table 13 Training and test results for sinus rhythm with only unipolar EGMs.

| **Training (%)** | | **AUC** | **Accuracy** | **F1-score** | **PPV** | **Sensitivity** | **Specificity** |  |
| --- | --- | --- | --- | --- | --- | --- | --- | --- |
| Logistic | 0.640  [0.611-0.670] | | 64.7±11.1 | 18.7±6.9 | 11.1±4.5 | 63.6±18.1 | 64.8±11.8 |  |
| KNN | 0.689  [0.669-0.709] | | 76.4±3.2 | 22.2±7.0 | 14.1±4.8 | 56.8±18.4 | 77.8±3.2 |  |
| SVM | 0.671  [0.653-0.688] | | 73.1±10.4 | 23.0±8.0 | 14.7±6.2 | 61.0±10.9 | 73.9±11.1 |  |
| RF | 0.729  [0.709-0.749] | | 69.8±9.4 | 22.3±7.1 | 13.6±5.2 | 67.7±12.0 | 70.0±9.7 |  |
| **Test (%)** | **AUC** | | **Accuracy** | **F1-score** | **PPV** | **Sensitivity** | **Specificity** | |
| Logistic | 0.594 | | 55.7 | 13.5 | 7.5 | 63.6 | 55.3 | |
| KNN | 0.751 | | 76.5 | 24.5 | 14.8 | 70.3 | 76.8 | |
| SVM | 0.733 | | 75.4 | 22.1 | 13.3 | 64.4 | 76.0 | |
| RF | 0.821 | | 71.9 | 23.9 | 14.0 | 81.4 | 71.4 | |

Table 14 Training and test results for LV-pacing with all EGMs.

| **Training (%)** | **AUC** | **Accuracy** | **F1-score** | **PPV** | **Sensitivity** | **Specificity** |  |
| --- | --- | --- | --- | --- | --- | --- | --- |
| Logistic | 0.677  [0.651-0.703] | 66.6±10.9 | 24.1±10.0 | 15.4±7.3 | 64.2±14.1 | 66.8±11.6 |  |
| KNN | 0.713  [0.697-0.730] | 71.0±4.5 | 26.7±9.1 | 17.1±6.9 | 66.9±13.7 | 71.4±4.7 |  |
| SVM | 0.673  [0.658-0.688] | 72.9±9.7 | 27.8±9.7 | 18.7±8.0 | 61.7±9.2 | 73.8±10.6 |  |
| RF | 0.752  [0.733-0.772] | 70.2±9.4 | 28.5±11.5 | 18.5±9.1 | 71.9±10.6 | 70.1±9.8 |  |
| **Test (%)** | **AUC** | **Accuracy** | **F1-score** | **PPV** | **Sensitivity** | **Specificity** | |
| Logistic | 0.657 | 74.1 | 23.5 | 14.8 | 57.8 | 75.3 | |
| KNN | 0.725 | 67.8 | 22.4 | 13.4 | 67.6 | 67.8 | |
| SVM | 0.740 | 75.2 | 26.7 | 16.8 | 65.7 | 75.9 | |
| RF | 0.729 | 71.0 | 22.9 | 14.0 | 62.7 | 71.6 | |

Table 15 Training and test results for LV-pacing with bipolar EGMs.

| **Training (%)** | **AUC** | **Accuracy** | **F1-score** | **PPV** | **Sensitivity** | **Specificity** |  |
| --- | --- | --- | --- | --- | --- | --- | --- |
| Logistic | 0.668  [0.649-0.686] | 61.8±9.5 | 22.1±8.4 | 13.7±6.2 | 67.3±11.0 | 61.4±10.3 |  |
| KNN | 0.628  [0.617-0.640] | 58.7±4.6 | 19.7±6.5 | 11.9±4.5 | 63.9±10.4 | 58.3±5.0 |  |
| SVM | 0.587  [0.570-0.604] | 62.9±8.2 | 19.9±6.8 | 12.4±4.9 | 57.3±11.1 | 63.5±8.9 |  |
| RF | 0.677  [0.657-0.695] | 63.6±9.2 | 23.0±7.9 | 14.2±5.8 | 66.3±9.3 | 63.2±10.0 |  |
| **Test (%)** | **AUC** | **Accuracy** | **F1-score** | **PPV** | **Sensitivity** | **Specificity** | |
| Logistic | 0.623 | 62.0 | 19.4 | 11.4 | 66.7 | 61.6 | |
| KNN | 0.631 | 58.9 | 17.3 | 10.1 | 62.7 | 58.6 | |
| SVM | 0.618 | 54.8 | 17.5 | 10.0 | 69.6 | 53.7 | |
| RF | 0.678 | 69.3 | 21.4 | 13.0 | 60.8 | 69.9 | |

Table 16 Training and test results for LV-pacing with unipolar EGMs.

| **Training (%)** | **AUC** | **Accuracy** | **F1-score** | **PPV** | **Sensitivity** | **Specificity** |  |
| --- | --- | --- | --- | --- | --- | --- | --- |
| Logistic | 0.656  [0.629-0.682] | 68.2±11.1 | 24.3±9.6 | 15.6±7.0 | 62.8±16.6 | 68.5±12.0 |  |
| KNN | 0.719  [0.700-0.737] | 71.5±5.0 | 27.0±11.0 | 17.6±8.8 | 67.0±14.7 | 71.9±5.2 |  |
| SVM | 0.657  [0.638-0.676] | 74.0±10.6 | 28.1±10.9 | 19.4±9.3 | 59.6±10.6 | 75.2±11.6 |  |
| RF | 0.761  [0.745-0.777] | 72.2±8.4 | 30.2±9.5 | 19.6±7.6 | 72.6±8.8 | 72.1±8.8 |  |
| **Test (%)** | **AUC** | **Accuracy** | **F1-score** | **PPV** | **Sensitivity** | **Specificity** | |
| Logistic | 0.665 | 76.9 | 25.0 | 16.1 | 55.9 | 78.5 | |
| KNN | 0.750 | 70.7 | 24.4 | 14.8 | 68.6 | 70.9 | |
| SVM | 0.708 | 77.3 | 28.2 | 18.0 | 64.7 | 78.3 | |
| RF | 0.792 | 72.2 | 26.1 | 16.0 | 71.6 | 72.2 | |

Table 17 Training and test results for RV-pacing with all EGMs.

| **Training (%)** | **AUC** | **Accuracy** | **F1-score** | **PPV** | **Sensitivity** | **Specificity** |  |
| --- | --- | --- | --- | --- | --- | --- | --- |
| Logistic | 0.676  [0.649-0.703] | 69.6±12.3 | 23.9±8.7 | 15.1±6.2 | 63.9±13.5 | 69.9±13.2 |  |
| KNN | 0.698  [0.679-0.715] | 74.8±4.8 | 24.4±8.0 | 15.8±5.8 | 59.1±15.9 | 76.0±5.0 |  |
| SVM | 0.680  [0.660-0.700] | 74.2±9.1 | 25.4±8.7 | 16.8±7.1 | 60.0±13.0 | 75.4±9.8 |  |
| RF | 0.739  [0.719-0.758] | 71.3±9.4 | 26.2±8.7 | 16.5±6.5 | 68.7±10.0 | 71.4±10.0 |  |
| **Test (%)** | **AUC** | **Accuracy** | **F1-score** | **PPV** | **Sensitivity** | **Specificity** | |
| Logistic | 0.612 | 51.1 | 14.9 | 8.4 | 66.7 | 50.1 | |
| KNN | 0.656 | 72.2 | 20.3 | 12.4 | 55.0 | 73.4 | |
| SVM | 0.653 | 57.4 | 16.2 | 9.3 | 64.2 | 57.0 | |
| RF | 0.671 | 63.3 | 18.9 | 11.0 | 66.7 | 63.1 | |

Table 18 Training and test results for RV-pacing with bipolar EGMs.

| **Training (%)** | **AUC** | **Accuracy** | **F1-score** | **PPV** | **Sensitivity** | **Specificity** |  |
| --- | --- | --- | --- | --- | --- | --- | --- |
| Logistic | 0.675  [0.656-0.695] | 63.0±8.2 | 20.7±5.9 | 12.4±3.9 | 66.6±10.4 | 62.8±8.6 |  |
| KNN | 0.607  [0.595-0.619] | 60.8±4.3 | 16.7±4.5 | 9.9±3.1 | 55.9±10.0 | 61.2±4.6 |  |
| SVM | 0.614  [0.602-0.625] | 62.6±7.8 | 18.5±4.9 | 11.1±3.6 | 60.0±7.9 | 62.8±8.6 |  |
| RF | 0.675  [0.660-0.690] | 64.5±8.0 | 20.7±5.7 | 12.5±3.9 | 65.0±9.3 | 64.4±8.5 |  |
| **Test (%)** | **AUC** | **Accuracy** | **F1-score** | **PPV** | **Sensitivity** | **Specificity** | |
| Logistic | 0.555 | 51.4 | 13.7 | 7.7 | 60.0 | 50.8 | |
| KNN | 0.568 | 59.9 | 14.4 | 8.3 | 52.5 | 60.5 | |
| SVM | 0.557 | 55.5 | 14.6 | 8.3 | 59.2 | 55.2 | |
| RF | 0.545 | 47.1 | 12.7 | 7.1 | 60.0 | 46.2 | |

Table 19 Training and test results for RV-pacing with unipolar EGMs.

| **Training (%)** | **AUC** | **Accuracy** | **F1-score** | **PPV** | **Sensitivity** | **Specificity** |  |
| --- | --- | --- | --- | --- | --- | --- | --- |
| Logistic | 0.696  [0.669-0.720] | 70.4±10.5 | 24.6±8.8 | 15.6±6.3 | 64.7±14.0 | 70.7±11.1 |  |
| KNN | 0.681  [0.663-0.700] | 74.8±3.5 | 23.3±9.1 | 15.1±6.6 | 55.6±16.7 | 76.3±3.6 |  |
| SVM | 0.662  [0.640-0.683] | 71.7±9.1 | 23.4±7.8 | 15.0±6.1 | 59.8±13.0 | 72.6±9.6 |  |
| RF | 0.735  [0.716-0.754] | 71.3±9.3 | 26.1±8.5 | 16.5±6.4 | 68.4±9.5 | 71.5±9.7 |  |
| **Test (%)** | **AUC** | **Accuracy** | **F1-score** | **PPV** | **Sensitivity** | **Specificity** | |
| Logistic | 0.622 | 52.9 | 16.3 | 9.2 | 71.7 | 51.6 | |
| KNN | 0.623 | 73.6 | 18.8 | 11.7 | 47.5 | 75.4 | |
| SVM | 0.628 | 56.9 | 16.2 | 9.3 | 65.0 | 56.3 | |
| RF | 0.658 | 65.2 | 17.7 | 10.4 | 58.3 | 65.7 | |
